# Channing’s Notes on Anæmia

**Published:** 1842-10-15

**Authors:** 


In the last number of the New England Quarterly Journal, are some interesting cases of anæmia, or, as the author insists on writing it, Anhæmia, chiefly in reference to the puerperal state. Well known as the symptoms of anæmia are, it will be from time to time mistaken by men who have never learned the distinctive characters of disease, and can never persuade themselves that headache and violent action of the heart and arteries do not always require depletion. The more we bleed the worse the patient becomes, and the temporary relief which the loss of blood sometimes produces, is very soon followed by an increase in the distressing symptoms.

Generally speaking, anæmia is not dangerous. But it becomes so when complicated with any severe disturbance of the system, as the puerperal state; or when it is followed by a grave disorder, it is often indirectly the cause of death.

As the importance of the subject cannot be too strongly impressed upon our readers, we give Dr. Channing's remarks relative to some points in the history of the disease.

*Diagnosis.*—Anhæmia after delivery may be confounded with that state which follows immediately, or soon after, uterine hemorrhage. In the first or immediate state, the surface may resemble anhæmia; while some of the symptoms of *reaction* are still more nearly like it. A very little observation will show that the *color* of the skin after hemorrhage, is wholly unlike the *no color* of anhæmia. The skin, in the first place, may be blanched; but you see at once that something of its natural hue remains. It is not *red* in any sense of the word, but in this circumstance alone does it look like that of anhæmia. There is *complexion*. Then again the face especially, and to this are these remarks confined, after hemorrhage, is sunken, wanting its natural expression; showing in the suddenness of the exhaustion, how sudden has been the action of the cause which has produced it.

*Reaction.*—After uterine hemorrhage, this sometimes is excessive, and along with the continued pallor, its symptoms nearly resemble anhæmia. The principal of these symptoms is the violent action of the heart and arteries, and the sounds which accompany this action. In the *brain* we have sounds, which may be thought to imitate those which accompany anhæmia. They differ, however, in this. They are called a *beating*, or rather thumping in the head; and are referred to various parts of the head, the temples especially, not to the ears; they are *felt* rather than *heard;* and I have never known them to be compared to the distinct noises that are so painfully annoying in anhæmia. The least motion of the head increases the trouble almost to agony. The woman gets no sleep, and manifests extreme and general distress. So with the *heart.* Its action is tumultuous, audible to the patient, and is increased by the least motion.

Now how soon do all these troubles, incident to an excessive functional effort, give place to a controlled and salutary degree of *reaction* after *hemorrhage?* Sometimes again it passes into the truly morbid, even to the production of *peritonitis puerperarum*, or puerperal fever. So often has this been noticed, that judicious observers tell us, that in an epidemic invasion of that disease, we are not to look for exemption from it, in the profuse, nay, dangerous hemorrhages which may have attended labor. We may even be called on to practise active depletion in just those cases in which we might *a priori* have supposed it least likely to have been demanded. I might here show how prompt is sometimes this *reaction*, how salutary soever it may be, to pass into threatening local inflammation. Witness a case reported some time since in the London Medical and Physical Journal, of transfusion of blood done to prevent death from uterine hemorrhage, in which in a day or two afterwards it became necessary to apply *leeches* for a threatening *phlebitis*, which had attacked the arm in which the transfusion had been done. Thus it is that simple *reaction* from *loss of blood* differs entirely from anhæmia. Every day that passes makes the safety greater in the one state, while time only developes more and more rapidly the fatal result of the other.

*Diseases of the Heart and Arteries.*—I have already given at some length a case, that of S. H., which was regarded as organic disease of the heart, and in which bloodletting had been so frequently practised to save life. In that case anhæmia would seem to have been produced, or certainly not benefited, by the depletion for the supposed disease. Examination showed the error of the diagnosis. The heart was perfectly normal in its structure. I know, or rather have heard of a similar mistake, after a most careful *diagnosis;* and have before me the record of a supposed veritable aneurism of the *aorta*, which turned out to be no aneurism at all, i. e. at the *autopsy.* These were both cases of anhæmia. In these instances the disease was chronic, at least had existed much longer than is common with the disease which they seemed so exactly to imitate. The diagnosis of the more usual forms of anhæmia, which are strictly *acute*, or are speedily terminated, cannot be very difficult. The palpitation of the heart, and pulsation of the arteries, may be never so tumultuous, still there is over and above all these, so much obviously threatening disease, with occasionally, I acknowledge, most extraordinary contradictions, that a careful observer cannot but see that the apparently most pressing symptoms are in reality the least so ; that death is surely coming, but to which event those symptoms in themselves would hardly be supposed to lead. And how rare is it for those symptoms to be dwelt on by the patient ? Nothing has been to me more striking, I had almost said startling, than the perfect serenity, the emphatic prophecy that life will soon cease, and the composure with which that event has been looked to, as these have been manifested by women, and by young women too, who have been just placed in the most important and interesting relation of life. You feel at the moment, and the thought never leaves you, that this mysterious malady has not its place in any one organ, an organ whose obvious lesion is after death to tell you the story of its cause. The whole material organism is disturbed, diseased, while the immaterial, the spiritual, has gained new power in the very midst of all this physical confusion.

But how is anhæmia distinguished from the organic diseases just named ? I have named one means of diagnosis in what I have just written. Then the symptoms given before, aid the question. You do not find in it any of the local or general symptoms which mark an organic disease soon to be fatal. The characteristic œdema of the face, of the feet, of the ancles, the ascites, the deficient urine, the thirst, the febrile commotion, the emaciation, the difficulty of any particular *decubitus*, especially the strongly expressed demand for an elevated position of the body ; all these are wanting in anhæmia. In the genuine disease, attacks are not paroxysmal, at least not so as in simple nervous heart, or the graver organic lesions of that organ. There is no emaciation. At least I remember but one case in which this was very striking, viz. in the protracted one of the young man which stands first in the series. And how strong is the contrast between the marked serenity and absence of complaint, even where the disturbance of the circulation is greatest, and that deeply expressed, and visible anxiety, and even pain almost amounting to agony, which attends the severer paroxysms of heart disease. I cannot but notice this, now that I am attending a young person with such disease in its worst form, whose life for years has been an almost continuous suffering, and who, now apparently at the close of life, is conscious of nothing but physical misery, and yet is daily occupied with the delusive hope of recovery.

*Chlorosis.*—In its chronic state anhæmia may imitate chlorosis, and by excessive depletion for some of the severe symptoms of the latter, the former I think may be produced. I have given a probable case of this kind. In its connection with the *puerperal* state, however, whether acute or chronic, I hardly think such an error of diagnosis can arise. I, however, have said quite enough of the disease itself, and of its imitations, to make it unnecessary to point out in detail, or even at all, in what it differs from chlorosis.

*Prognosis.*—The character of this is easily to be gathered from what has been said in every page of this paper. The hourly and daily persistency of the same symptoms, with the as strongly marked failure of all the powers of life, and the unceasing progress to death which almost every case has made, tells us what the *prognosis* should be. The unfavourable character of this gets new force from this single and simple fact, that in the two cases of anhæmia which are reported to have recovered, there was no such change produced by any portion of the treatment, as authorized those who attended the cases to decide in any degree on what these recoveries depended.

*Treatment.*—The last question does much to settle the questions which the treatment of anhæmia involves. The dissections which have been reported in this paper, and it would have been easy to have added to them, have thus far shed too little light on the nature of the disease to guide us in its treatment. These teach us how fatal a disease may be, the individual instances of which may have so strong a resemblance to each other as almost to seem to depend on some specific cause, and still leave no marks behind. The most which has been done by treatment, has been to attempt to answer the most obvious indications; and in the midst of universal physical prostration, with perfect mental vigor, to assist what power remains in sustaining the functions on which living depends. The question of *transfusion* has often occurred to me. But of what possible benefit would be such a supply of blood? What might not the effect be of filling almost empty vessels with a fluid so unlike that which already circulates in them, and which *their own functions* have produced? In a disease so fatal some risk might be incurred. But is transfusion an operation which our present knowledge of it would authorize? If safe in itself, however, might not time be gained by the operation, for such functional changes to occur as would supply healthful blood?

---

*Circular.*—It gives us great pleasure to comply with Dr. Oppenheim's request by inserting the following circular.

The frightful conflagration which visited Hamburg in the beginning of last May has not spared the premises of the Medical Union, whose Library, the fruit of twenty years assiduous collection, exists no more! Such a loss cannot be repaired by pecuniary contributions. Complete series of a great number of German, French, English, American, and Indian journals and works, rare editions of the older authors, a multitude of ancient and modern medical and chirurgical enclycopædias and lexicons in various languages, scarce and curious prints, &c., are not only lost, but are no longer procurable by purchase; while many hundred volumes of old dissertations, classified according to subjects, cannot be replaced in any manner. In this strait the Medical Union earnestly requests advice, not only from its foreign members, but from all its medical brethren, where and in what manner it may once more gradually acquire possession of a library at the least possible expenditure of money. Any communication on this subject, in post paid letters or through the medium of the booksellers, addressed to "The Directors of the Hamburg Medical Union," or to the undersigned, will be received with the sincerest thanks. The editors of the medical journals are requested kindly to give insertion to this notice in their respective publications.

F. W. Oppenheim, M. D.

*Hamburg*, May 16th, 1842.

